# Progress in Surgery

**Published:** 1898-08-06

**Authors:** 


### PROGRESS IN SURGERY.

### RECTUM AND ANUS.

At a meeting of the Medical Society of London Mr. Bryant read a paper on "Rectal Surgery."[1] He regards pruritus ani as always a symptom of some local rectal trouble, even if it be only some slight irritating rectal secretion or a small rectal polypus.

Abscesses about the rectum readily burrow, and should be opened early in every case, otherwise they are apt to end as complicated fistulæ. In examining fistulæ, the internal orifice should always be made out if possible; this examination may be aided perhaps by injecting the fistula with milk. After the first 24 hours it is unnecessary to daily plug the wounds if tags of skin have been carefully removed.

Bryant draws attention to perforating ulcers of the rectum, which on perforation give rise to a spreading infective cellulitis of the cellular tissue around, or to a recto-vesical fistula. He cites four such cases. Pain associated with so-called piles should give rise to the suspicion of a fissure or ulcer, and lead to careful examination.

The introduction of Kelly's tubes or proctoscopes has led to more complete examination of the rectum and sigmoid. Dr. James Tuttle has recorded his experience.[2] He finds the tubes particularly useful in the diagnosis of early carcinoma, and in distinguishing atrophic and hypertrophic catarrh of the rectum. Numerous tubes of various sizes should be at hand. Imagination has perhaps exceeded discretion in diagnosis, one surgeon attributing melancholia to prolapse of the bowel, but, says the *Medical Record*, "the brain does not lie in the gut."

Attention has centred of late on inflammation of the rectum, and Dr. D. F. Talley has written a paper on "Chronic Proctitis."[3] He distinguishes two varieties (apart from their causes): (*a*) When there is ulceration and vegetation of the mucous membrane; and (*b*) when there is stenosing fibrosis of the submucous tissues. In the treatment careful dieting, complete rest, and stretching the sphincter are indicated. Under anæsthesia the ulcers are touched with a strong solution of nitrate of silver in most cases.

**Rectal Stricture.**—Rieder[4] has set himself the task of deciding (*a*) whether syphilitic stricture really exists; (*b*) why it is more common in women. He found inflammation and degeneration of the vessels in the contracting granular tissue, but only in the veins, not in the arteries. The hæmorrhoidal veins especially were attacked by obliterating endophlebitis. There were scattered miliary gummata. The veins are partly involved by constricting fibrous tissue and partly by cellular infiltration through their coats. In late stages there are few vessels remaining, and the stricture consists of elastic scar-tissue. The frequency of syphilitic stricture in women is explained by the fact that in them the pudendal veins communicate almost directly with the hæmorrhoidal.

**Hæmorrhoids.**—Dr. George Reinbach (Beiträge zur Klin. Chirurgie, Vol. XIX.) finds the old view—that hæmorrhoids consist of dilated veins—untenable. He concludes that they really consist in a new formation of young blood-vessels by germination from older vessel walls, and the consequent formation of cavernous tissue. Venous stasis has nothing to do with the affection. He gives four types: (*a*) germinating capillaries, (*b*) cavernous tissue, (*c*) venous angeioma, and (*d*) vessels with inflamed thickened walls.

In the diagnosis of hæmorrhoids, Dr. Bryant lays stress on the importance of giving the patient an enema beforehand.[5] He thinks excessive consumption of brown meats predisposes to the affection. Inflamed hæmorrhoids with prolapsus ani are best left alone for a time, and astringents and anodynes applied locally. Mr. Pridgin Teale is dissatisfied with the clamp and cautery for piles. He now prefers to excise piles individually, and to stitch up the cut mucous membrane with catgut. In a discussion which followed a paper by Dr. Parker Syms,[6] it was pointed out that, amongst other objections to Whitehead's operation, was the fact that it removed the sensory apparatus of the linea pectinata, and therefore precipitate defecation was apt to occur.

**Primary Tuberculosis** of the rectum can only be diagnosed by the aid of the microscope.[7] It is often curable by local treatment, but the operations, comprising curetting and cautery, must be early and repeated.

**Rectal Aspiration.**—In an editorial of the *Lancet*,[8] reference is made to some of these remarkable cases in which, by a physical effort, the patient can inspire a large quantity of air per anum and expel it again, thus producing the sounds of thunder, the trombone, the quacking of a duck, and even going so far as to smoke a cigarette!

**Cancer of the Rectum.**—The advantages of Kraske's sacral operation are not fully established. The mortality is great. Even in Kraske's recent series of 80 cases there was an immediate mortality of 20 to 25 per cent., and fully 25 per cent. more died of recurrence from six months to twelve years afterwards.

The majority were in male patients, and of the cylindrical celled variety, appearing to take origin from the mucous membrane itself, and not from any embryonic tissue in most cases. Kraske holds that heredity plays an important rôle. The course is usually rather slow, but diagnosis is rarely early. A careful digital examination under chloroform will alone secure positive evidence in suspicious cases. Kraske says the high position of a growth is no contra-indication to operation. On the whole, the performance of Kraske's operation by surgeons in general should be very carefully considered before being adopted, owing to its high mortality and the requisite manipulative experience. Dr. Desforges-Mereil believes that the perineal method, with modifications, is the best for cancer of the rectum.[10] Dr. Joseph M. Mathews draws the following conclusions: That the high operation, that total extirpation of the rectum, and that Kraske's operation are not to be recommended, and that opening the peritoneum is to be deprecated. All malignant growths within reach of the finger should be extirpated by the circular method from below.

[1] Lancet, Jan. 29, 1898. [2] New York Med. Rec., April 9, 1898. [3] Columbus Med. Jour., Mar. 1898. [4] Archiv. für Klinische Chirurgie, Band lv., S. 730. [5] Lancet, Mar. 5, 1898. [6] New York Med. Jour., Feb. 12, 1898. [7] Therapeutic Gazette, Feb. 15, 1898, p. 95. [8] Lancet, June 25, 1898, p. 1,764. [9] Internat. Med. Mag., Dec., 1897. [10] New York Med. Rec., Jan. 22, 1898.

